# Eight proteins play critical roles in RCC with bone metastasis via mitochondrial dysfunction

**DOI:** 10.1007/s10585-015-9731-4

**Published:** 2015-06-27

**Authors:** Jiang Wang, Xiaolin Zhao, Jun Qi, Caihong Yang, Hao Cheng, Ye Ren, Lei Huang

**Affiliations:** Department of Orthopedics, Tongji Hospital, Tongji Medical College, Huazhong University of Science and Technology, Jie Fang Ave 1095#, Wuhan, 430030 China; Department of Information Science, School of Mathematical Sciences and LMAM, Peking University, Beijing, 100871 China

**Keywords:** Renal cell carcinoma, Bone metastasis, Mitochondria dysfunction, System biology, Proteomics

## Abstract

Most kidney cancers are renal cell carcinomas (RCC). RCC lacks early warning signs and 70 % of patients with RCC develop metastases. Among them, 50 % of patients having skeletal metastases developed a dismal survival of less than 10 % at 5 years. Therefore, exploring the key driving proteins and pathways involved in RCC bone metastasis could benefit patients’ therapy and prolong their survival. We examined the difference between the OS-RC-2 cells and the OS-RC-2-BM5 cells (subpopulation from OS-RC-2) of RCC with proteomics. Then we employed Western-blot, immunohistochemistry and the clinical database (oncomine) to screen and verify the key proteins and then we analyzed the functions and the related pathways of selected key proteins with system biology approaches. Our proteomic data revealed 26 significant changed spots (fold change <0.5 and >1.9, P < 0.05) between two cells. The Western blotting results validated for these identified spots were consistent with the proteomics’. From the public clinical database, 23 out of 26 proteins were connected with RCC metastases and 9 out of 23 with survival time directly (P < 0.05). Finally, only 8 out of 9 proteins had significantly positive results in tissues of RCC patients with bone metastasis compared with primary tumor (P < 0.05). System biology analyzing results showed these eight proteins mainly distributed in oxidative phosphorylation which indicates that mitochondria dysfunction played the critical role to regulate cells metastasis. Our article used a variety of experimental techniques to find eight proteins which abnormally regulated mitochondrial function to achieve a successful induction for RCC metastasis to bone.

**Electronic supplementary material:**

The online version of this article (doi:10.1007/s10585-015-9731-4) contains supplementary material, which is available to authorized users.

## Introduction

In adults, renal cell carcinoma (RCC) is the most common type of kidney cancer, responsible for approximately 80–85 % of cases [1]. Around 270,000 cases of kidney cancer are diagnosed yearly and 116,000 people die from the disease in the world. Approximately 90 % of all kidney cancers are RCC [2]. RCC is characterized by a lack of early warning signs, which results in a high proportion of patients with metastases [3] and it is a common group relatively resistant to radiation therapy and chemotherapy as well. 70 % of patients with RCC develop metastases during the course of their disease and patients with metastatic RCC have a dismal survival of less than 10 % at 5 years [4, 5]. Among them, approximately 50 % of patients with metastatic RCC have skeletal related diseases development and more than 35 % dying with RCC have skeletal metastases [5]. Patients with bone destruction from metastatic RCC often have devastating complications including pain, impending or actual fractures, is neurologic compromise from spinal lesions, anemia, and complications exacerbated by immobilization such as hypercalcemia [6]. Currently, the incidence of RCC is increasing but the mortality rate has not improved significantly, most likely because currently available therapies for bone metastatic disease have little efficacy [6–8]. Thus, bone metastasis in RCC is one of the major causes of increased morbidity and eventual mortality and it is the future focus for therapy to RCC patients as well. Now, the priority is to find the key driving proteins for RCC with bone metastasis, which plays the key roles in improving for RCC with bone metastasis patients’ prognosis and developing preventive/therapeutic strategies.

**Fig. 1:**
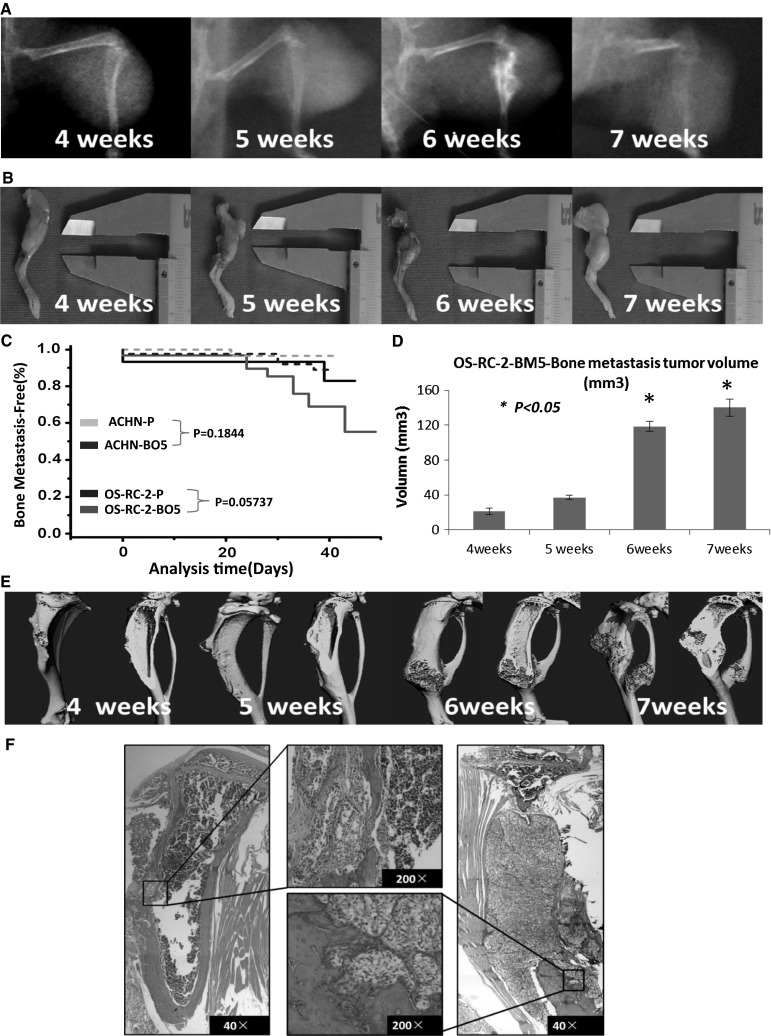
**a** The osteolytic lesion taken by X-ray shown from from 4th to 7th week in nude mice tibias. **b** The tumor sizes of bone metastasis measured by vernier caliper from 4th to 7th week. **c** Kaplan–Meier curves showing the bone metastases activity of the parental (ATCC)ACHN and OS-RC-2 cells population, various in vivo selected subpopulations. Metastasis was scored as the time to first appearance of a visible bone lesion by X-ray imaging. **d** The volume data showed as column chart. Tumor volume was calculated by formula: volume = (width)^2^ × length/2. **e** Is Micro-CT examination for mice legs with bone metastasis, after fixed by 4 % buffered formalin. **f** The images of haematoxylin and eosin (HE) staining on bone metastasis lesions. The original magnification is ×40 and ×200. *Asterisk* means there is a significant difference between 6th or 7th week and 5th week (P < 0.05)

Previously we established the bone-seeking (ACHN-BO) subpopulation from the human RCC cell line ACHN in nude mice. We already showed the phenotypic changes from ACHN parental cells (ACHN-P) to bone-seeking (ACHN-BO) that could be a reason for RCC cells to develop and accelerate bone metastases. However, the molecular mechanisms are not fully understood [9]. To fully explore the pathogenesis of RCC bone metastasis, we use another RCC cell line OS-RC-2 to develop the animal model like before, which has higher incidence of bone metastases and shorter tumorigenic time according to our previous method with a little variation [9] (Fig. 1c). We also established the bone-seeking cell clones (OS-RC-2-BO5). After investigating the proliferation and invasion between the OS-RC-2-BM5 and OS-RC-2 cells, we compared the expression of proteins between these two groups cell by 2D gel electrophoresis (2-DE) and got the 100 different protein expressions. Among these proteins, 26 significant changes proteins which involve 3 down-regulated and 23 up-regulated were selected. We checked the clinical relevance of these 26 proteins from the public clinical data (www.oncomine.com) and RCC patients’ tissues. Finally we selected 8 proteins that most probably play an indispensable role for bone metastasis of RCC.

For a better understanding the interaction, function of these eight proteins and related pathway involved, we employed the system biological approaches to analyze them and surprisingly found these 8 proteins are most likely to induce RCC bone metastases by abnormally regulating the mitochondrial function.

In this article, we used a variety of experimental techniques, from animal models to cellular and molecular levels, from in vitro to in vivo and combining with the clinical data, to confirm that 8 proteins screened from 2-DE were pretty active in the formation of RCC bone metastases. Furthermore, we utilized system biology approaches to analyze the proteins’ function, protein–protein interaction (PPI) and signaling pathway to confirm the mitochondria dysfunction may play a critical role for metastasis, which may shed light on the clinical therapies for bone metastases of RCC patients.

## Materials and methods

### Cells and animals

OS-RC-2 cells, a human renal cancer line, were obtained from China Center of Type Culture Collection (CCTCC), Wuhan (China). For selection in vivo, 5–8 weeks old nude mice (BALB/c-nu) were obtained from the Experimental Animal Center of Huazhong University of Science and Technology (Wuhan, China). All animals were housed under pathogen-free conditions and maintained according to the guidelines of the Committee on Animals of Huazhong University of Science and Technology.

### Cell growth, invasion and cell cycle assay

Cell growth and invasion assay were detected with MTT and Trans-well migration assay (Corning). The flow cytometry was used to detect the cell cycle. All the experimental procedures can be found in [9].

### In vivo selection of OS-RC-2-BM5

The detailed experimental methods can be seen in previous publications [10]. Here, in brief, nude mice were anesthetized and injected into the left ventricle with 0.1 ml of OS-RC-2 cells (1 × 10^6^ cells). The osteolytic lesions in limbs were detected by radiography (every 1–2 days starting from the beginning) and after sacrificing, the affected limbs were separated from the body. Tumor cell suspension was collected. These subpopulations (OS-RC-2-BM) were again inoculated into the left heart ventricle. Following five passages of in vivo selection, a highly bone metastatic cell line, OS-RC-2-BM5, was obtained for further experiments. All animal experiments were approved by the Tongji Hospital affiliated Huazhong University Animal Ethics Committee.

### Radiological and micro-computed tomography analysis of osteolytic lesions and samples histology

Using a Digital Faxitron small animal X-ray cabinet (Faxitron X-Ray, Wheeling, IL, USA) at 35 kV tube voltage, 0.3 mA current and 4 s exposure time, Osteolytic lesions in limbs were monitored. The image analysis software (Analysis Software Imaging System GmbH, Germany) was used to quantify the lesion area in limbs. The μCT system is a cone beam tomograph (Scanco Medical, Switzerland) with a microfocus X-ray tube providing a 10-μm focal spot. 3D-image surface reconstructions were performed using the μCT software. Additional sagittal view of tibial lesion were performed on a desktop computer using customized software for image analysis [11, 12]. The tissue sections of 4 µm thick were cut and stained with hematoxylin and eosin (H&E) and standard protocols were used as before [9]. Samples and tissue sections from patients were observed by immunohistochemistry and stained with DAB [13]. The primary antibodies resource can be seen in Supplementary 4 (Table 2).

### Protein preparation

After removing the culture medium from the cells, the adherent cells were washed once with PBS followed by an additional short rinsing with ice-cold double distilled H_2_O to remove remaining proteins. The tissue culture dish was quickly frozen with liquid nitrogen, which was allowed to warm up to room temperature and 1 ml lysis buffer and was added with PMSF protease inhibitor (Thermo scientific and Sigma-Aldrich Co). Transferred suspension to fresh tubes, frozen with liquid nitrogen and sonicated in an ice-cold ultrasonic bath for 30 min. Subsequently, the lysate was centrifuged at 13,000 rpm for 5 min at 4 °C. 2-D Quant Kit (GE Healthcare, USA) was used to measure the protein content of supernatant.

### Two-dimensional gel electrophoresis (2DE)

Protein samples, 150 mg for silver-stained gels and 500 mg for colloidal Coomassie-stained gels, were precipitated and resuspended. The samples experienced rehydrated and followed by the IEF procedure on 24 cm non-linear immobilized gradient strips (GE Healthcare). Electro focusing was carried out. Next, proteins were reduced at 20 °C for 20 min and followed by alkylation. The second dimension was carried out on SDS PAGE gels and 8 W/gel at 15 °C. After fixed, analytical gels were stained using silver nitrate. [14].

### Spot detection and quantitation

The Delta 2D software version 4.0 (Decodon company) was used to evaluate the Gel. A spot detection was performed with the Delta 2D software for the fused image of all time points [15]. Spot intensities were firmed and normalized. The more details can be seen [16].

### Western blot analysis

Equal quantities of protein were processed for Western blotting [17]. Each sample was denatured, electrophoresed, and transferred onto a PVDF membrane. After blocking the membrane, blots firstly were incubated in specific antibodies and then secondary antibodies following the manufacturer’s instructions. The results of Western blot were quantified with Gene Snap soft-ware (Syngene, America). The primary antibodies were listed at Supplementary 4 (Table 2).

### Clinical pathological assessment

Totally, 57 patients’ samples were used to analyze expressions of screened proteins. Among them, 14 cases are normal kidney, 16 cases are from primary RCC and 27 samples are derived from bone metastasis lesions of RCC patients. All samples were gotten from tissues of surgical resection from Tongji Hospital of Wuhan China between March 2001 and December 2013. The methods of fixation, decalcification, section and staining were followed as above using common protocols. The informed consent was obtained from all individual participants included in this study.

### System biology approach

The public clinical database was from Oncomine V4.5. The proteins’ function, protein–protein interaction (PPI) and related pathway analysis relied on IPA (Ingenuity), STRING and DAVID (gene function classification tool).

### Statistical methods

All statistical data in the samples were showed as mean ± SD. Statistical analysis was performed using SPSS 13.0 (SPSS, USA). *T* test was used to compare the difference between two samples. In Kaplan–Meier curves, the discrepancy between two samples was compared with Chi square test. Differences were considered to be statistically significant when the P value was <0.05.

## Results

### The establishment of OS-RC-2-BM

In the first cycle of inoculation, 13.33 % (4/30) of the transplanted nude mice with OS-RC-2cells inoculated through the left ventricle had tumor clones in bone and lung. The remaining 26.67 % (8/30) showed lung metastasis alone and 60 % (18/30) of the transplanted mice were observed for 10 weeks without bone and other organs’ metastasis and tumor-related death. The bone metastasis tumor clones were dissected and used for culture. In the second cycle, 20 % (6/30) of mice had bone metastasis and 40 % (12/30) mice appeared both skeleton and lung metastasis. We continued to repeat the experiments by injecting mice with higher chance of bone-metastasis cells. Until the fifth cycle, the highest percentage of bone metastasis in the transplanted mice was observed, 53.33 % (16/30). Most of the bone metastasis sites were spine and the four limbs. We named the fifth cycled bone metastasis cells as OS-RC-2-BM5 with histological analysis confirmation.

Most animals developed aggressive osteolytic bone metastasis monitored by radiography (Fig. 1a). The tumor sizes were also measured by caliper (Fig. 1b). The incidence of bone metastasis by parental OS-RC-2 and OS-RC-2-BM5 cells compared with ACHN cells in Fig. 1c. Tumor volume was calculated with results shown in Fig. 1d [18]. At the 6th and 7th week, tumor volume significantly increased to 118.32 ± 5.87 and 140.92 ± 9.716 mm^3^ compared with 5th week (P < 0.05). Parts of legs were sent to micro-CT (Fig. 1e) examination. In addition, parts of tumor mass were processed into (HE) staining (Fig. 1f), which showed the destruction of bone tissue and a large group of tumor cells gathering at the site of osteolysis. The results from radiography, micro-CT and H&E histology examination tell us that the OS-RC-2-BM5 cells derived from renal cancer cell lines because of clear cell morphology and more characteristic for bone-seeking than OS-RC-2 cells, which also caused bone tissue swollen, reduced trabecular, thin bone cortical and mainly with osteolytic destruction. The observed extensive bone destruction was similar to those noted in clinical settings which mainly occurred in cancellous bone like side of tibia and femur, Spine and even pelvis in RCC patients with metastasis.

### The proliferation and invasion of OS-RC-2-BM5 cell line in vitro

**Fig. 2:**
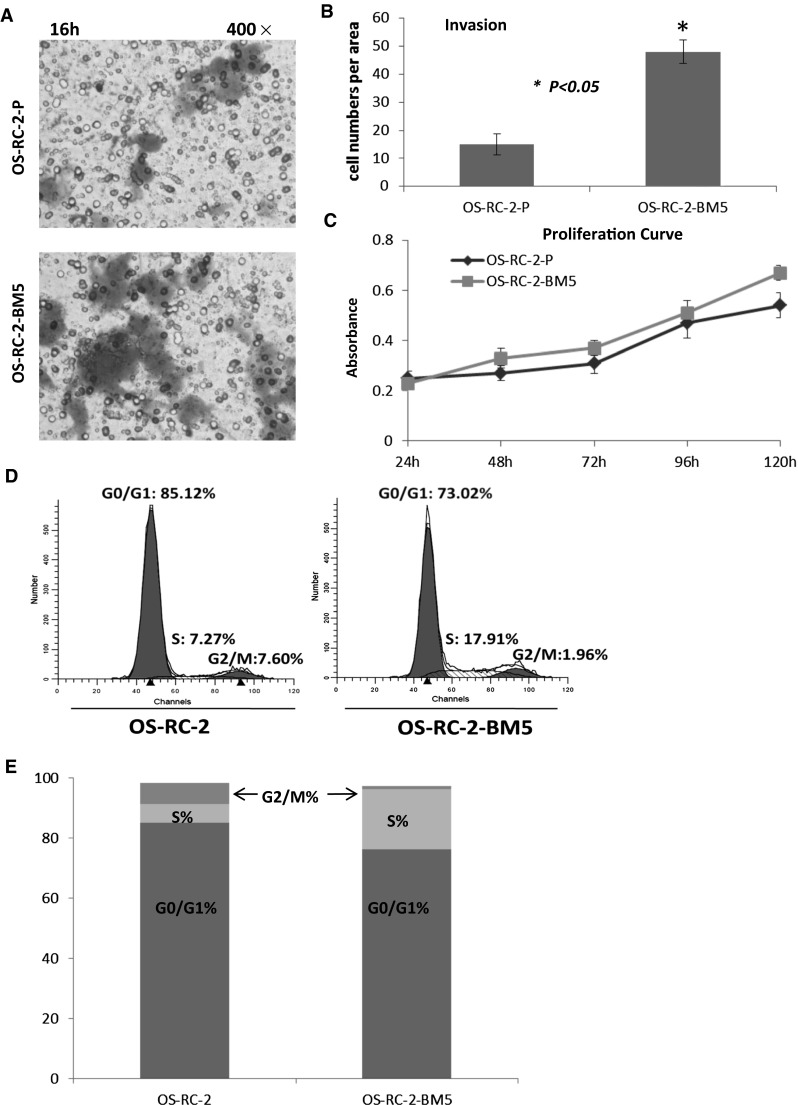
**a** The transwell migration assay to investigate the invasive ability of OS-RC-2-P and OS-RC-2-BM5 cells after 16 h incubation. **b** The number of transmembrane cells for OS-RC-2-P and OS-RC-2-BM5 cells showed as column chart. *Asterisk* means the transmembrane number of OS-RC-2-BM5 cells is pretty higher, comparing to OS-RC-2 cells, P < 0.05. **c** The proliferation curve which was got by MTT assay at time point 24, 48, 72, 96, 120 h respectively. **d** The flow cytometry analyzing cell cycle. **e** Comparison of cell cycles on two cell lines

First of all, we used Trans-well invasion assay to investigate the invasive ability of OS-RC-2 and OS-RC-2-BM5 cell lines. After 16 h, we washed the top matrigel and stained the penetrated cells as seen in Fig. 2a. There are more OS-RC-2-BM5 went through the matrigel than OS-RC-2 (P < 0.05) in Fig. 2b. Next, we tested the cell proliferation by MTT assay. Data from 24, 48, 72, 96, 120 h time point were collected for proliferation curve. The more quickly proliferation in OS-RC-2-BM5 cells was observed than OS-RC-2-P cells (Fig. 2c). In addition, the results from flow cytometry treated with propidium iodide (PI) showed us the proportions of two cells in S stage and G2/M stage was notably different. There are 13.32 ± 1.56 % OS-RC-2 cells on S phase plus G2/M phase, but 24.31 ± 2.13 % OS-RC-2-BM5 cells on S phase and G2/M phase in Fig. 2d, e, which means the more OS-RC-2-BM5 cells in proliferation stage.

### 2-DE results and Western blot verification

**Fig. 3:**
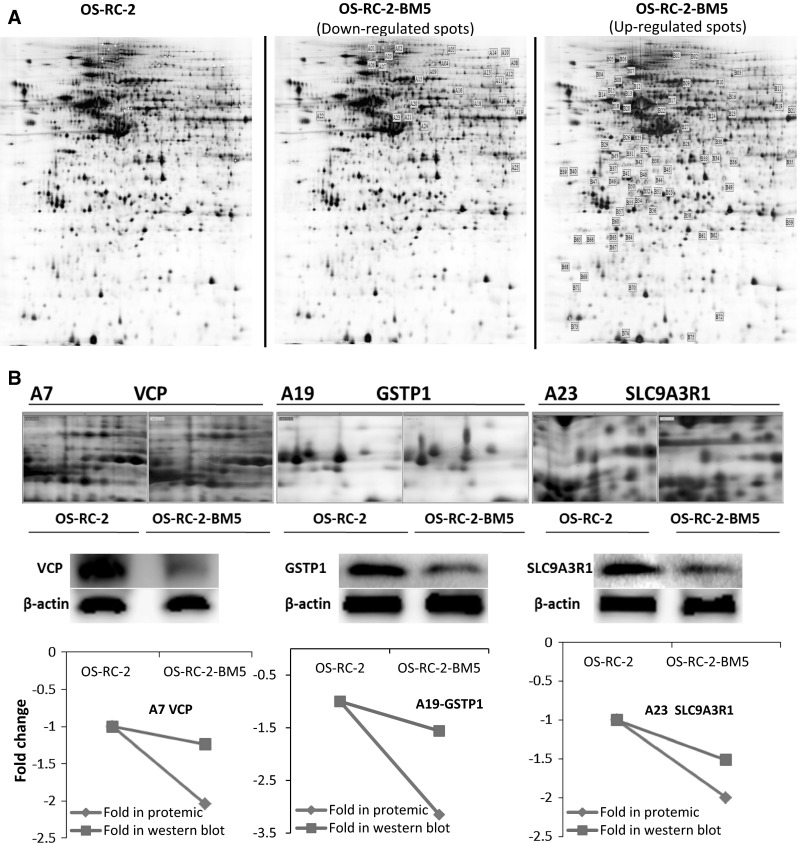

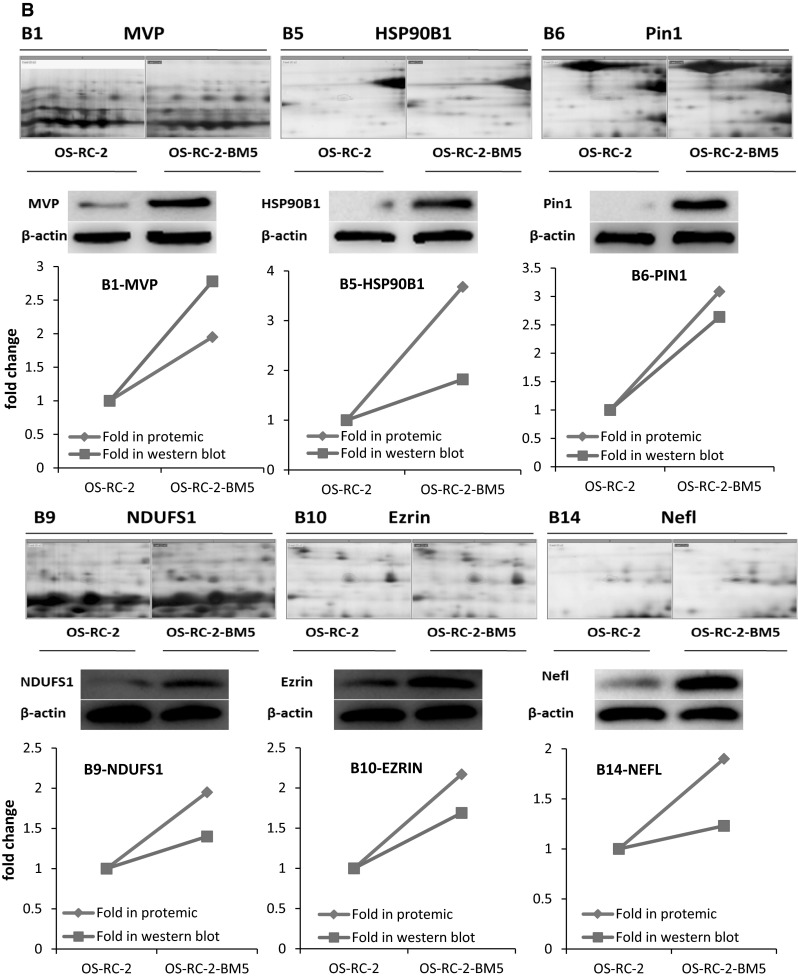

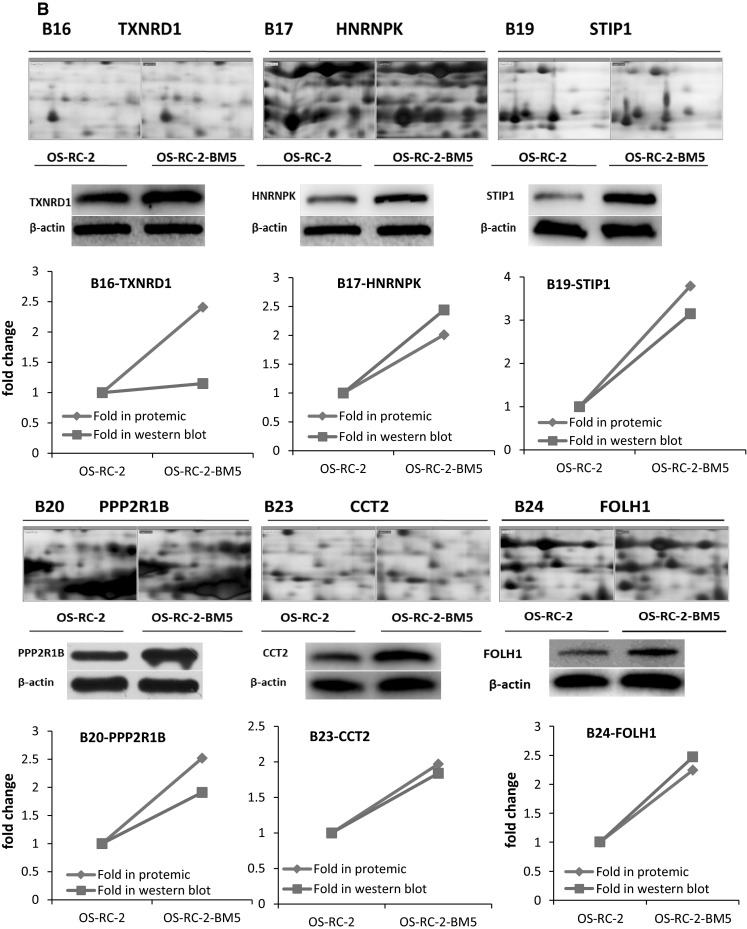

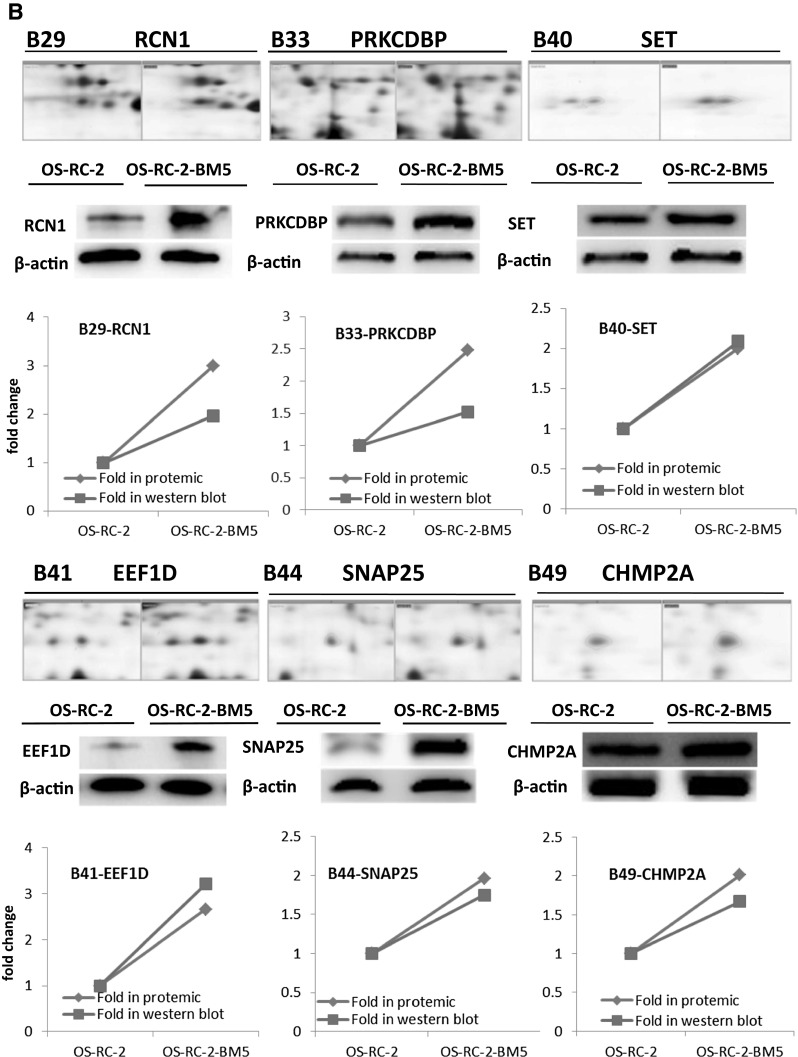

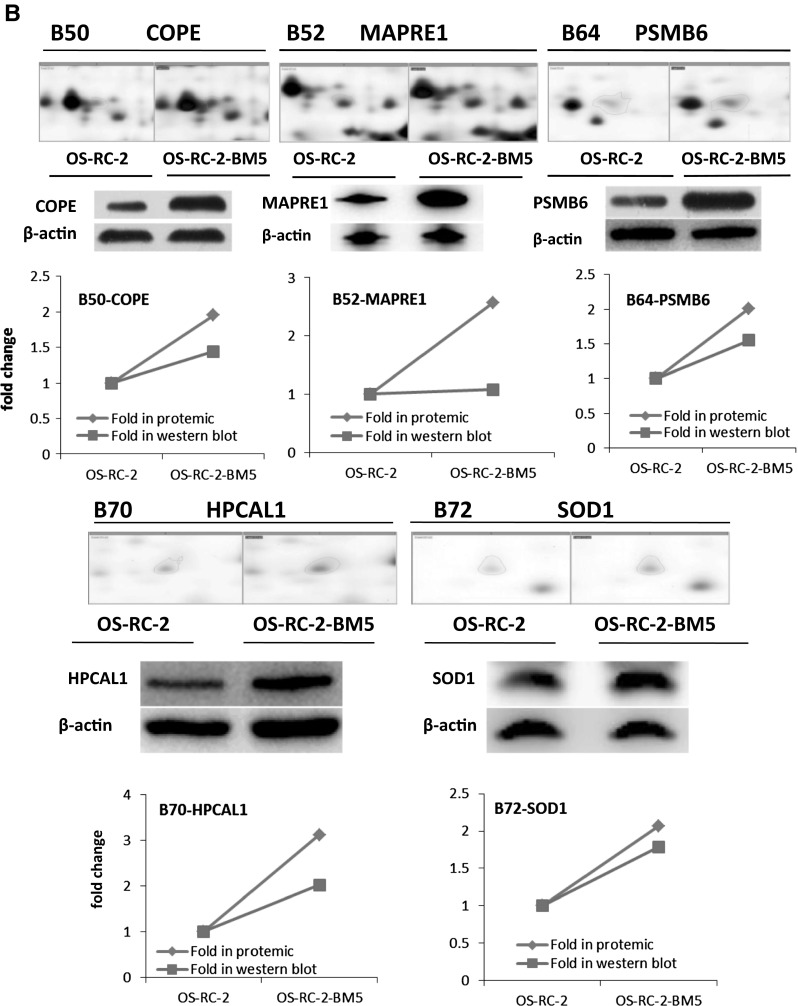
**a** The 2-DE gel showing the different spot intensities between OS-RC-2 and OS-RC-2-BM5. Among these altered proteins, 25 protein spots (P < 0.05) were noticeably down-regulated in the OS-RC-2-BM5 group (*middle panel*) and 75 protein spots (P < 0.05) were significantly up-regulated in the OS-RC-2-BM5 group on the right panel. **b** Results of quantitative Western blotting experiments performed for 26 proteins (change fold <0.5 and >1.9). The magnified images of protein spots from the 2-DE gels were shown on the upper part of each panel and the Western blot replicate results list below. The *line charts* showing the protein levels based on 2-DE and Western blot results in the *bottom of panel*

In our study, crude proteins were extracted from OS-RC-2 and OS-RC-2-BM5 cells separately. More than 800 protein spots were detected and 100 altered spots displayed differentiation. The differences in spot intensities between two cells were analyzed in Fig. 3a. Among them, 25 protein spots (P < 0.05) were noticeably down-regulated and 75 protein spots (P < 0.05) were noticeably up-regulated in the OS-RC-2-BM5 on the right panel (Fig. 3a). 4 of 25 protein spots were down-regulated with fold change <0.5 and 26 of 75 spots were up-regulated with fold change >1.9. After further analysis to exclude the replicate identifications, the final identification of 3 down-regulated and 23 up-regulated proteins were allowed. All of the protein spots were successfully identified with confidence interval % (CI %) values >95 % and the matched proteins were obtained from the IPI database. The description on 3 + 23 proteins can be seen in Supplementary 5 (Table 3). The positions of identified spots are displayed by accession numbers on a fused gel image in Fig. 3a.

To validate the results of the proteomics analysis, quantitative Western blot assay were performed for 26 proteins. The alteration of proteomics and Western blot analysis of 26 proteins in two cells: The magnified images of protein spots from the 2-DE gels were shown on the upper and the Western blot replicate results list below. The line charts show the protein levels based on 2-DE and Western blot results (Fig. 3b). The comparison of Western blot with those of proteomics screening shows the corresponding protein expressions, which validated our proteomics screening method.

### Genes expression and patients prognosis

**Fig. 4:**
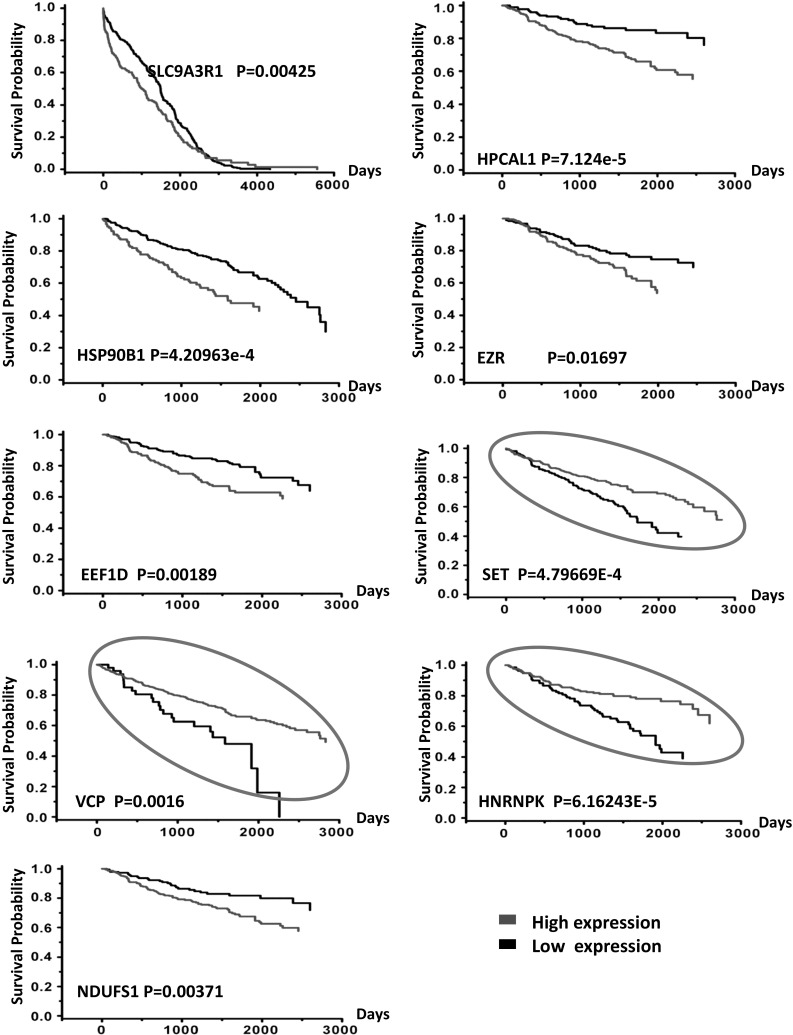
The prognostic signature on Kaplan–Meier curve showing 9 out of 26 genes significantly affected survival time of RCC patients when their expressions were high or low. These data originated in public clinical dataset. Gene expression was estimated by average as standard, expression higher than it was called high expression and the opposite ones named low expression. The K–M curves showed that patients with EEF1D, NDUFS1, HPCAL1, EZR, SLC9A3R1, HSP90B1 genes low expression survived longer than those with high expression. Conversely, the patients with high level VCP, SET and HNRNPK genes had a better prognosis than cases with low expression (*marked with circle*). *Red line* represents high expression and *black line* means low expression. P value is measured with log rank-test. (Color figure online)

We next searched these genes/proteins expression in public clinical datasets to see whether expressions of genes have any clinical relevance. TCGA Renal2, etc. 6 database were employed. TCGA Renal2 dataset including the maximum sample size were used for evaluation of prognosis. Results showed 23 out of 26 genes expressed higher in RCC patients with M1 than M0 (P < 0.05), originated in at least one database (Supplementary 2). 8 out of 23 genes (EEF1D, EZR, HNRNPK, HPCAL1, SET, SLC9A3R1, VCP and NDUFS1) showed this trend even in more than two databases. Additionally, these 8 genes also showed consistent trend: enhanced expression abided by rising tumor stages and grades. From Kaplan–Meier curve (Fig. 4), 9 out of 23 genes/proteins significantly affected survival time of RCC patients. Here, the K–M curves showed that Patients with EEF1D, NDUFS1, HPCAL1, EZR, SLC9A3R1, HSP90B1 genes low expression can survive longer than those with high expression. Conversely, the patients with high level VCP, SET and HNRNPK had a better prognosis.

Generally, high expression of the gene in patients with Metastasis indicates poor prognosis. Curiously, VCP, SET and HNRNPK genes expression status in RCC patients seems contrary to common sense between metastasis and prognosis. It must be mentioned that there are some limitations about M0, M1 stages in oncomine database without analyzing primary sites. Recent studies proposed that survival from metastatic cancer depends on the primary sites, which can interpret this phenomenon [19]. Thus, though there are 8 genes overlapped, it was still unable to discard any gene from these 9. But one thing is certain, looking through these proteins’ functions, it is not difficult to find, these proteins EEF1D, NDUFS1, SLC9A3R1, EZR, HNRNPK, HSP90B1, VCP either located at the membrane surface of organelle or were closely related to energy metabolism. It has been implicated that a number of cellular events are regulated during mitosis [20, 21].

### Immunohistochemistry results from patient samples

**Fig. 5:**
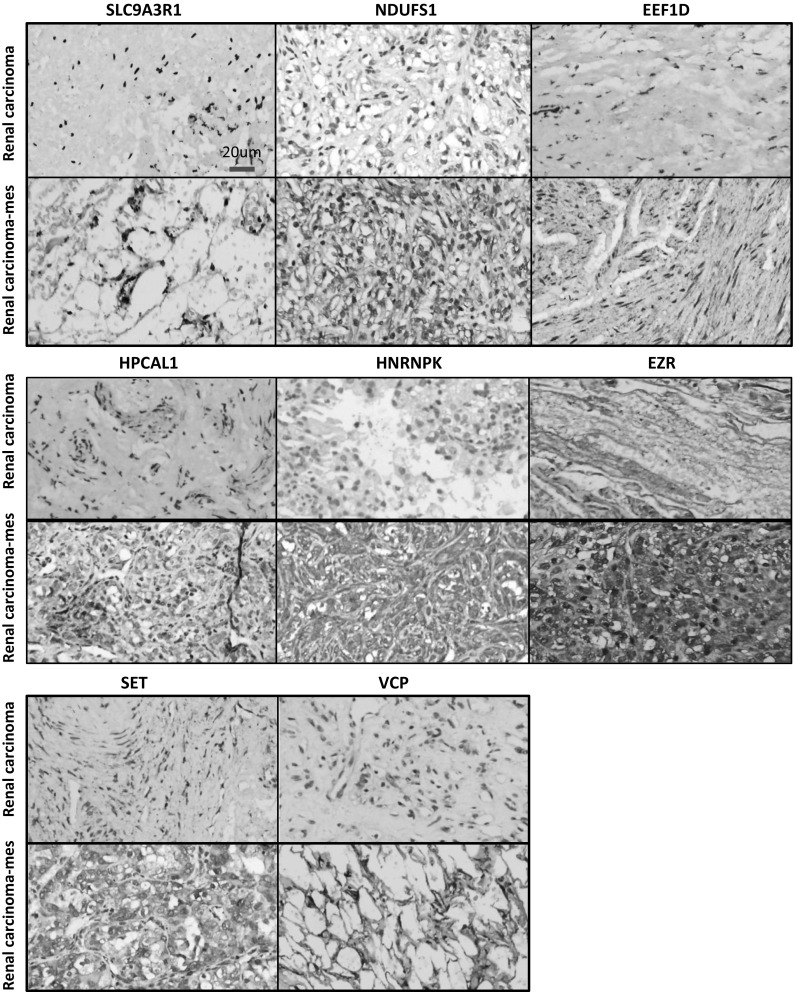
Results of immunohistochemistry DAB staining showing 8 genes/proteins expressed in samples primary RCC and secondary bone lesions of RCC patients. The eight positive results involved SLC9A3R1, NDUFS1, EEF1D, HPCAL1, VCP, SET, HNRNPK and EZR proteins. All of them were overexpressed in skeletal lesions of RCC patients comparing with primary RCC. *Scale bar* 20 µm

Subsequently, selected genes found to be differentially expressed at the protein levels were validated further by immunohistochemistry in RCC patient samples. We checked all 26 proteins expression in our clinical samples. Results showed that 8 genes detected their corresponding proteins had strong signals. Interestingly, these 8 proteins were covered by above nine. They are SLC9A3R1, VCP, NDUFS1, EEF1D, HPCAL1, EZR, SET, HNRNPK with low expression level in primary RCC but high in the bone metastasis lesions of RCC patients (Fig. 5).

### Protein identification and interactions

**Fig. 6:**
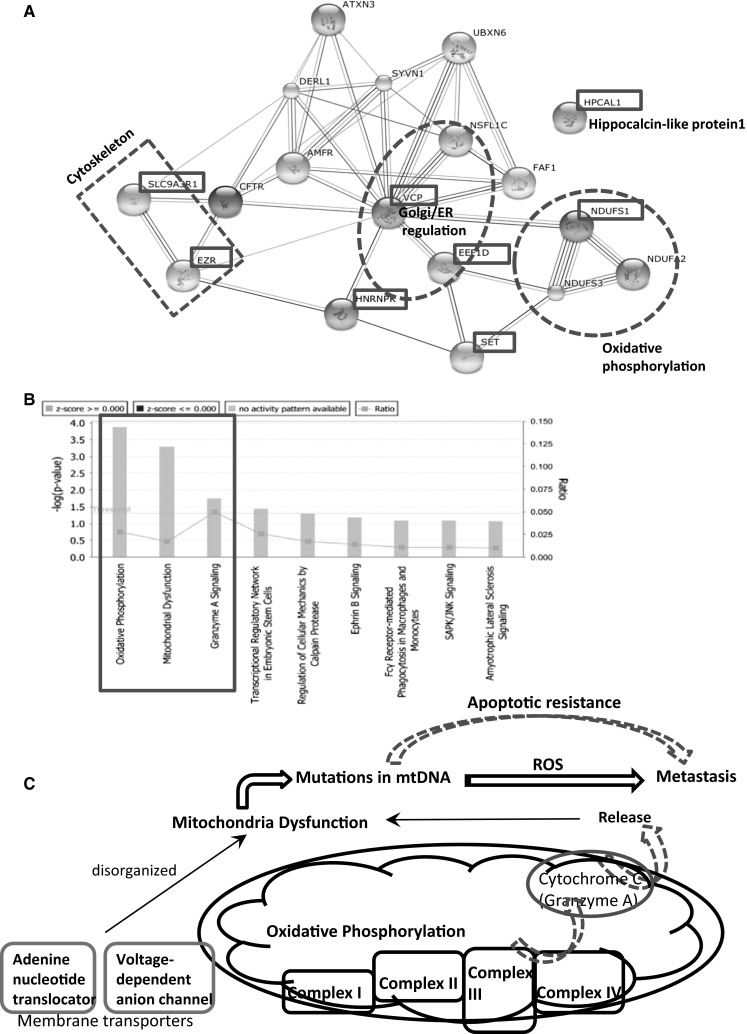
**a** The protein–protein interaction for the identified 8 proteins in STRING (10 necessary proteins/genes were added into the network so as to find the potential strong connection among them. The *red dotted* lines circled three main pathways. **b** The ingenuity pathway analysis (IPA) for all these 18 genes showing that oxidative phosphorylation, mitochondria dysfunction and granzyme A are the significantly activated pathways (fold change over 1.5, P < 0.05). **c** The possible mechanism related mitochondria functions: unspecific condition like inflammation, carcinogens, radiation (ionizing or ultraviolet), intermittent hypoxia, viral infections which is carcinogenesis in our study that damages a cell’s oxidative phosphorylation. Any of these conditions can damage the structure and function of mitochondria thus activating a respiratory chain changes (Complex I, II, III, IV) and also cytochrome c release. When the mitochondrial dysfunction persists, it produces genome instability (mtDNA mutation), and further lead to malignant transformation (metastasis) via increased ROS and apoptotic resistance. (Color figure online)

To classify identified proteins/genes, firstly we employed the DAVID (biology informatics software) to assigned the 26 proteins/genes from 2-DE into ten different categories in which the metabolism category was the largest and together with the categories like transcription-related, folding-associated proteins and cytoskeleton by which more than half proteins were covered (Supplementary 1A). Secondly, we used STRING (http://www.string-db.org), which is a database and a tool for predicting protein–protein interactions directly and indirectly. They are derived from the following sources: previous knowledge, high-throughput experiments, genomic context, conserved co-expression. From STRING (Fig. 6a), the potential strong connection can be seen, 10 necessary proteins/genes were added into the network except for identified 8 proteins. The official symbols and names of 18 genes corresponding to all proteins was list on Supplementary 3 (Table 1). Furthermore, the STRING analysis function showed these genes/proteins belong to different pathways: NDUFS1 NDUFS2 NDUFS3 is for oxidative phosphorylation process; EEF1D led VCP and NSFL1C to regulate Golgi/Endoplasmic Reticulum functions; SLC9A3R1 and EZR are important proteins on the cell membrane and cytoskeleton. The other proteins can be categorized into calcium-binding protein, protein kinase, glutamate carboxypeptidase and regulation of translational initiation and other functional proteins. Finally, we analyzed all these genes in the ingenuity pathway analysis (IPA). The result showed that oxidative phosphorylation, mitochondria dysfunction and granzyme A are the significantly activated pathways (fold change over 1.5) (Fig. 6b), which caused genes/proteins expression to change in RCC and increased their ability of invasiveness and metastasis. Accordingly, we summarized the possible mechanism that the primary renal carcinoma turned into particular bone metastasis cells in Fig. 6c.

## Discussion

Recently, the mechanistic understanding of how mitochondrial dysfunction contributes to cell growth and tumorigenesis is emerging. Most work focused on how dysfunctional mitochondria modulate cell cycle, metabolism, cell viability, gene expression and other established aspects of cell growth, etc. However, mitochondrial dynamics was modulated by key oncogenes and tumor suppressors through some key signaling pathways and that mitochondrial function vary between tumors and individuals and even the significance of these events for cancer have not yet been understood in detail [22]. Furthermore, some biochemical and morphological events recognized as characteristic features of apoptosis, the disruption of the inner mitochondrial transmembrane potential (ΔΨm) and the release of cytochrome c into the cytoplasm were considered as early events [23]. Thus, it is not difficult to infer the same events like interruption of inner mitochondrial transmembrane potential, the release of cytochrome c and reliance heavily on glycolysis to meet their metabolic demands happened in carcinogenesis. Coincidentally and interestingly, in our study, the 26 proteins differentiated with 2-DE were classified into 10 different categories, the metabolism category was the largest and covered more than 50 % proteins. This point was confirmed by IPA analysis as well (Supplementary 1B).

Next, we searched the public clinical microarray database (ONCOMINE) to further validate the 2-DE results. Results showed that 9 genes: SLC9A3R1, EEF1D, HPLAL1, NDUFS1, SET, EZR, HNRNPK, VCP, and HSP90B1 have close relation with RCC patients’ prognosis or metastasis. Taking into account the uncertainty of some data (set forth in Results) and more accurate identification for the key proteins, we collected 57 clinical samples and utilized the immunohistochemistry assay for all 26 proteins. Fortunately for us, the 8 key genes/proteins emerged significant positive signals in RCC osteolytic lesions, which can match with the expected outcomes and really covered by the above nine. So far, these 8 proteins/genes were verified with 2-DE, Western blotting, the public clinical microarray database and clinical patients’ sample detection, which implied they are very critical in RCC bone metastasis.

Subsequently, the 8 key genes functions were analyzed with system biology approaches. The oxidative phosphorylation process, Golgi/Endoplasmic Reticulum functions and some signal pathways related metabolism occupied the main reasons. Analogous results were found in metastatic breast cancer cells [24]. Thus, it had to make us convinced the respiratory chain involved in the metabolic disorders associated with the formation of bone metastasis in RCC patients. In fact, the role of respiratory chain represents the most basic mitochondrial functions. As core subunit of the mitochondrial membrane respiratory chain NADH dehydrogenase (Complex I), NDUFS1, NDUFS2, NDUFS3 were believed to belong to the minimal assembly required for catalysis. Deficiency and impairment of Complex I of the respiratory chain can cause many mitochondrial diseases [25–35]. Our studies showed NDUFS1 was overexpressed which directly reflected mitochondrial dysfunction. Recent researches also demonstrated that mitochondrial oxidative stress could actively promote tumor progression and increase the metastatic potential of cancer cells [36]. In view of these circumstances, it is reasonable to further recognize mitochondrial dysfunction was involved in skeleton metastasis of RCC.

In this research, VCP and EEF1D were found to express abnormally as well. Both them functioned to co-regulated Golgi-ER. VCP, the protein encoded by this gene belongs to a family that includes putative ATP-binding proteins. Likewise, EEF1D protein is responsible for the enzymatic delivery and it is a subunit of the elongation factor-1 complex as well. They has been implicated in a number of cellular events including regulation of mitochondria function [37–40].

Generally, reassembling of cytoskeletal proteins is an important feature of morphological changes during metastasis. Therefore, it is not surprising that SLC9A3R1 and EZR differentially regulated proteins, which belong to components of microfilaments, microtubuli and intermediary filaments [41, 42]. They are in the maintenance of cell architecture, adhesion, migration, differentiation, division, and organelle transport. The SLC9A3R1 protein localizes to microvilli, filopodia and membrane ruffles. It regulates and interacts with various proteins. Additionally, SLC9A3R1 also functions as linkers between cytoskeletal proteins and integral membrane [43]. EZR which also locates in microvilli, as cytoplasmic peripheral membrane protein, functions as an intermediate between the actin cytoskeleton and the plasma membrane. Particularly, it plays a key role in cell surface structure organization, adhesion and migration, and it has been implicated in various human cancers [44, 45]. The comparison of the results showed the group of up-regulated cytoskeleton proteins regulated concordantly mitochondria functions.

As we all known, cancer cells strongly up-regulate glucose uptake and glycolysis to give rise to increased yield of intermediate glycolytic metabolites and the end product pyruvate. At the same time, mitochondrial dysfunction precedes an aerobic glycolysis and can cause cancer cells genome instability and increased entropy and finally a series of cellular functions disorders [46, 47]. In addition, mitochondrial dysfunction leading to mtDNA mutations can enhance cancer metastasis through increased ROS and apoptotic resistance [48, 49].

Based on these evidence reviewed above, we depicted the potential mechanism in Fig. 6c. It showed how the normal cell changed to malignant behavior even metastasis potential: under unspecific carcinogenesis conditions like carcinogens, inflammation, radiation, viral infections, intermittent hypoxia which damaged a cell’s oxidative phosphorylation, the structure and function of mitochondria were damaged thus activating a respiratory chain changes (Complex I, II, III, IV) and also cytochrome c release. When the mitochondrial dysfunction persists, it produces genome instability (mtDNA mutation) and further lead to malignant transformation (metastasis) via increased ROS and apoptotic resistance. Hence, the impaired energy metabolism can be linked to invasion and metastasis if this impairment occurs in cells. [47].

Overall, in this paper, we elucidated protein profiles of OS-RC-2-P and OS-RC-2-BM5 cells. A number of proteins were altered in abundance and part of them was identified successfully. These proteins were involved in multiple functions and participated in a variety of biological processes in which dysregulation of mitochondrial function in RCC cells can push the formation of overt bone metastasis in clinic and therefore it is necessary for mitochondrial dysfunction to be taken a continued consideration as a therapeutic target on RCC bone metastasis in future research. Most importantly and hopefully, RCC patients can benefit from developing new antioxidant-based anti-cancer therapy to eliminate mitochondrial dysfunction and prevent or even reverse the formation of bone metastasis.

## Electronic supplementary material

Supplementary material 1 (PPTX 1376 kb)
**Supplementary 1** A. two-dimensional protein electrophoresis differentiated 26 proteins which can be assigned to 10 different categories (DAVID gene functional classification). B. IPA showing more proteins around metabolism category. Each node represents a protein and its association with other proteins is represented by a line. Nodes have different shapes to represent different molecule types. Detailed node information can be seen in it. The 21 proteins in network have a colored background: protein in red are up-regulated and proteins in green are down-regulated. Proteins with no background color were undetected in study but have been inserted by IPA to produce a highly connected network (www. Ingenuity.com). The direct interactions were represented by solid lines and dotted lines mean indirect associations. The isolated proteins were removed
**Supplementary 2** The clinical relevance was analyzed about positive genes/proteins expression status between RCC with and without metastasis via public clinical datasets (ONCOMINE). Results showed 23 out of 26 genes expressed higher in RCC patients with M1 than M0, originated in at least one database. The TCGA Renal2, TCGA Renal, Yang Renal, Jones, Zhao, Bittner, etc lab databases were employed. Patient data were chosen according to: the whole follow-up information, survival time record and with metastasis status

Supplementary material 2 (DOCX 74 kb)

Supplementary material 3 (DOCX 66 kb)

Supplementary material 4 (DOCX 107 kb)
