# Supplementary material for: Eight proteins play critical roles in RCC with bone metastasis via mitochondrial dysfunction
Source: Clin Exp Metastasis. 2015 Jun 27;32(6):605–22. doi: 10.1007/s10585-015-9731-4 (PMC4503866; doi:10.1007/s10585-015-9731-4)
Supplement: Supplementary file 1 — Supplementary material 1 (PPTX 1376 kb) Supplementary 1 A. two-dimensional protein electrophoresis differentiated 26 proteins which can be assigned to 10 different categories (DAVID gene functional classification). B. IPA showing more proteins around metabolism category. Each node represents a protein and its association with other proteins is represented by a line. Nodes have different shapes to represent different molecule types. Detailed node information can be seen in it. The 21 proteins in network have a colored background: protein in red are up-regulated and proteins in green are down-regulated. Proteins with no background color were undetected in study but have been inserted by IPA to produce a highly connected network (www. Ingenuity.com). The direct interactions were represented by solid lines and dotted lines mean indirect associations. The isolated proteins were removed Supplementary 2 The clinical relevance was analyzed about positive genes/proteins expression status between RCC with and without metastasis via public clinical datasets (ONCOMINE). Results showed 23 out of 26 genes expressed higher in RCC patients with M1 than M0, originated in at least one database. The TCGA Renal2, TCGA Renal, Yang Renal, Jones, Zhao, Bittner, etc lab databases were employed. Patient data were chosen according to: the whole follow-up information, survival time record and with metastasis status [file 10585_2015_9731_MOESM1_ESM.pptx]

## Slide 1
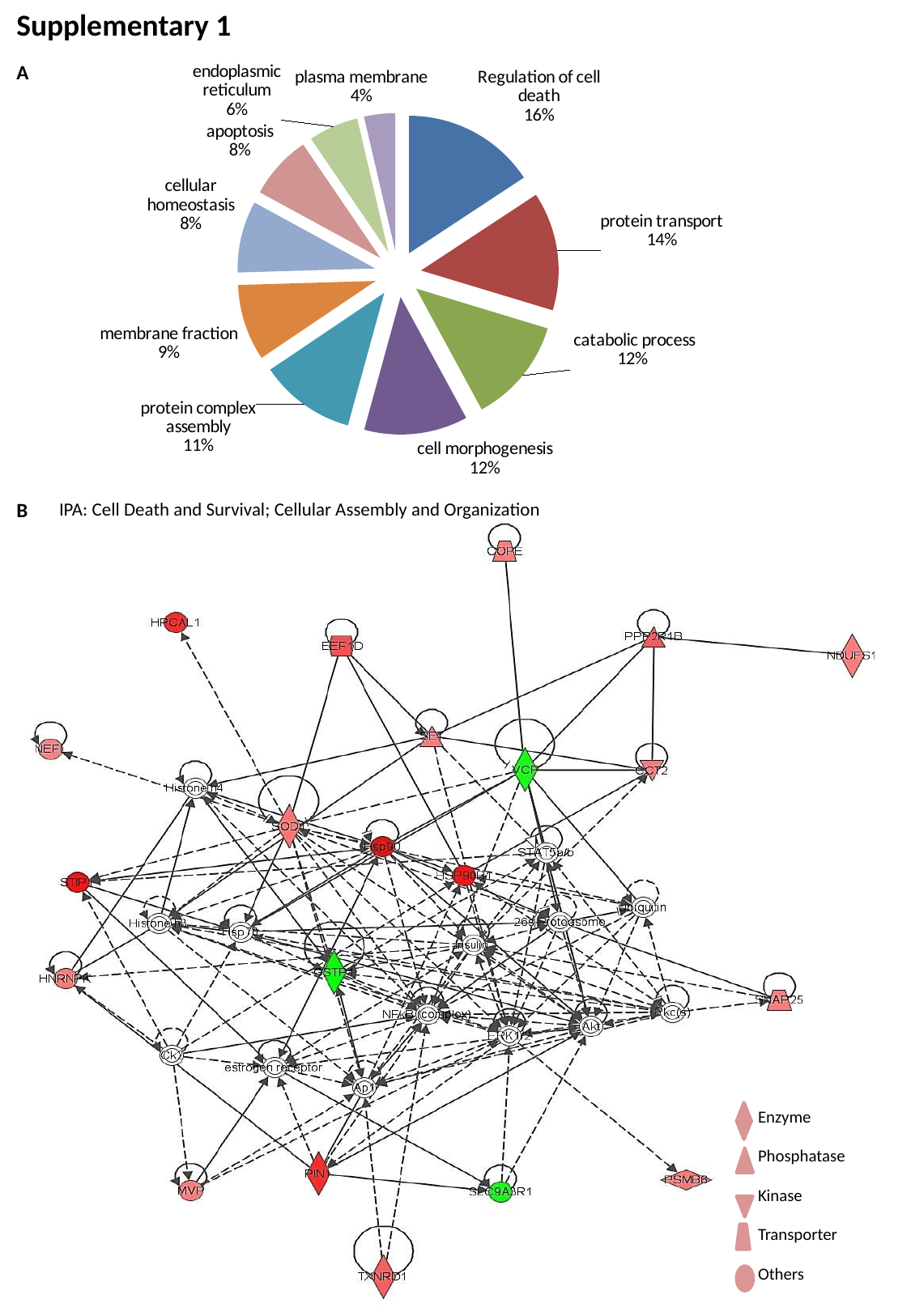

Supplementary 1
A
### Chart
| Category | |
|---|---|
| anti-apoptosis | 1.42 |
| protein transport | 1.26 |
|  catabolic process | 1.12 |
| cell morphogenesis | 1.1 |
| protein complex assembly | 1.02 |
| membrane fraction | 0.81 |
| cellular homeostasis | 0.76 |
| apoptosis | 0.68 |
| endoplasmic reticulum | 0.53 |
| plasma membrane | 0.33 |B
IPA: Cell Death and Survival; Cellular Assembly and Organization
Enzyme
Phosphatase
Kinase
Transporter
Others

## Slide 2
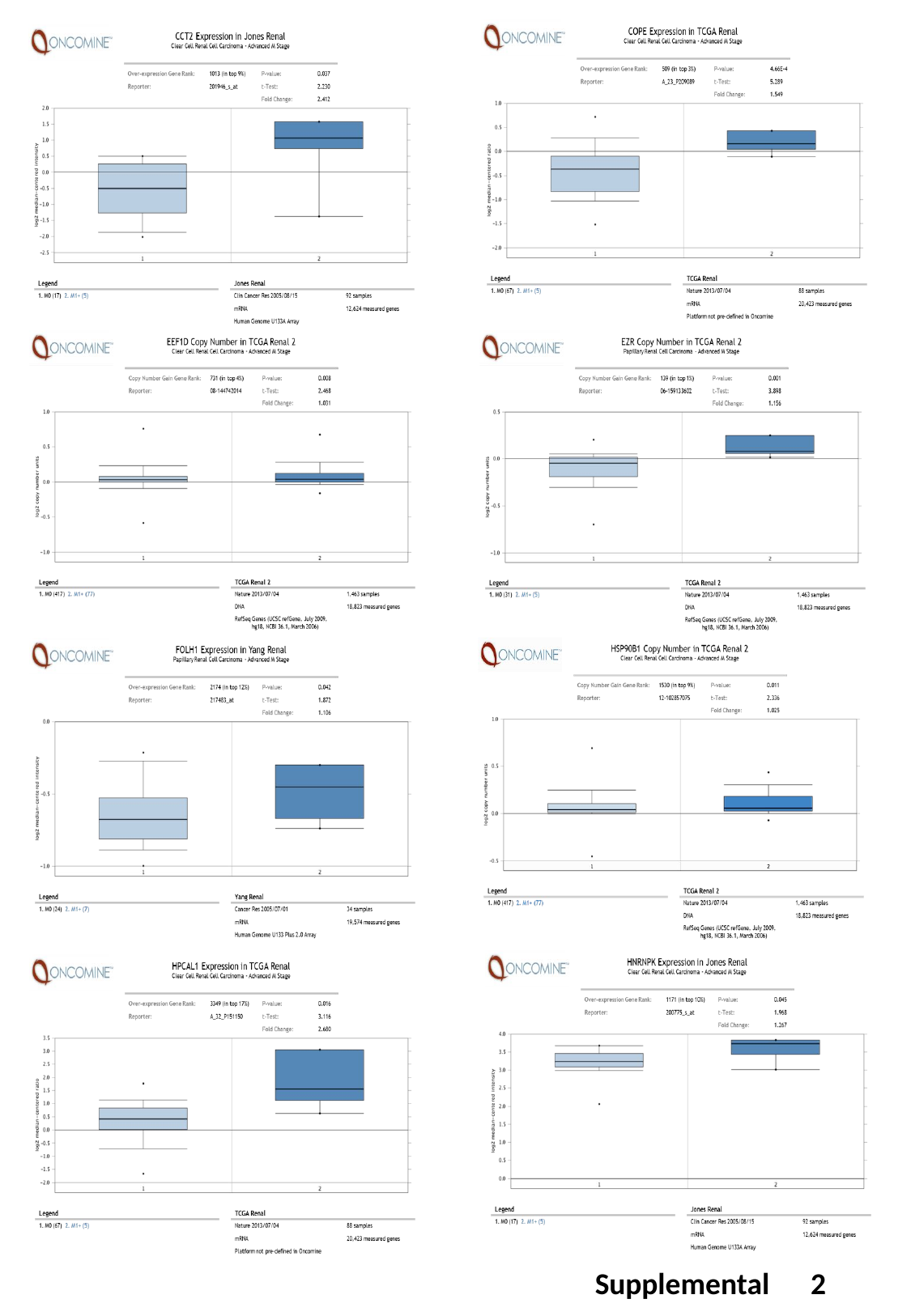

Supplemental 2

## Slide 3
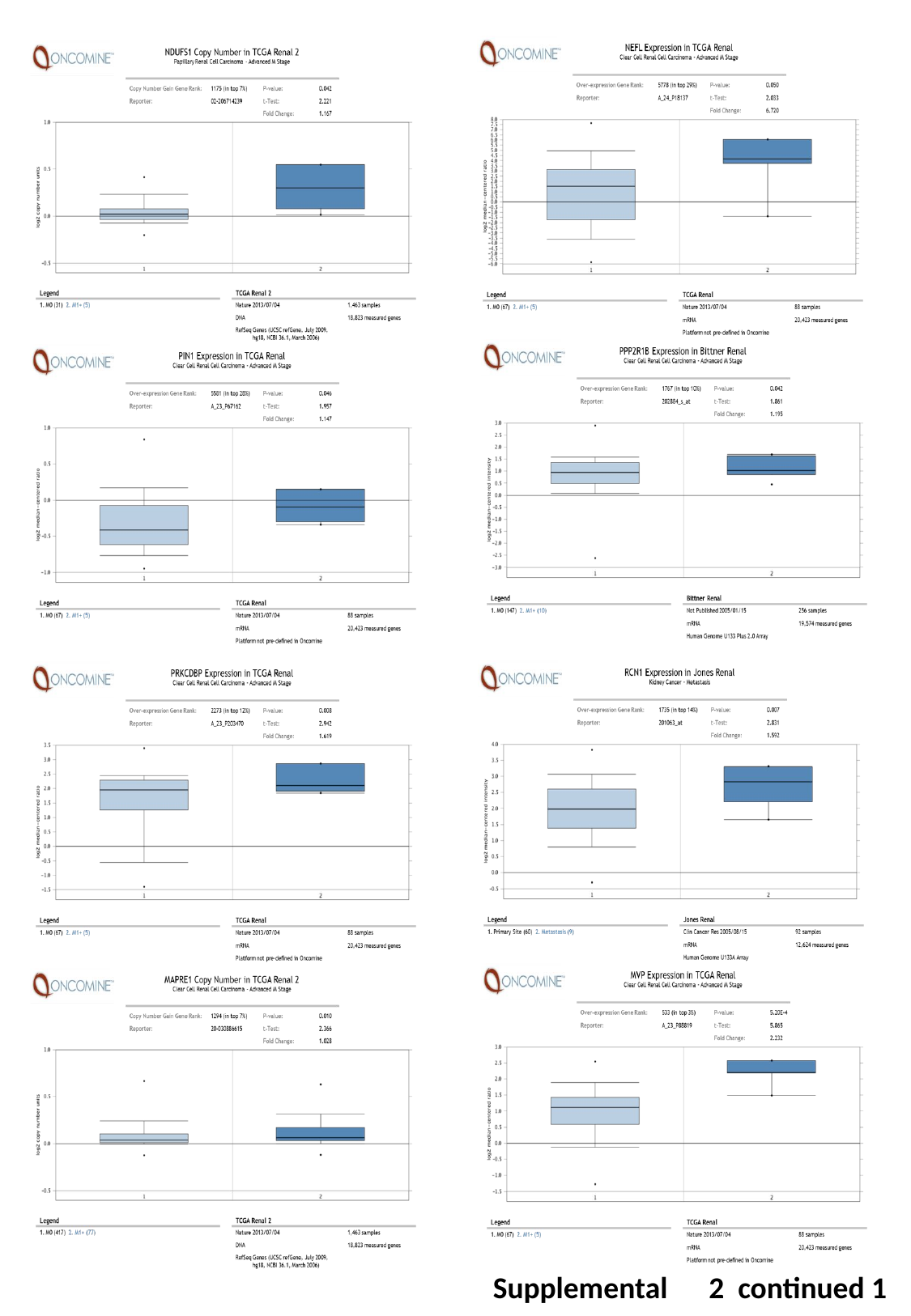

Supplemental 2 continued 1

## Slide 4
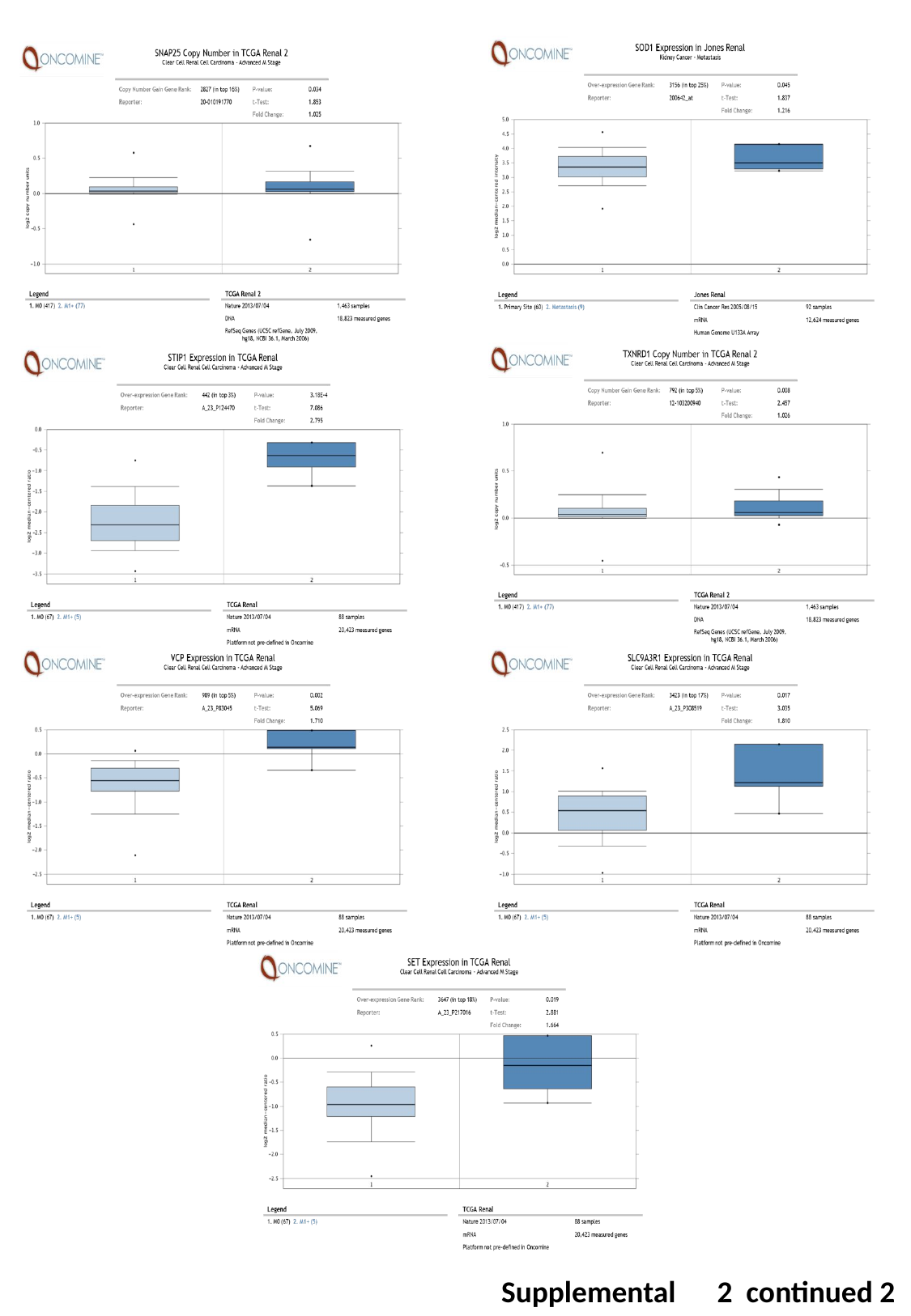

Supplemental 2 continued 2
