# Supplementary material for: Eight proteins play critical roles in RCC with bone metastasis via mitochondrial dysfunction
Source: Clin Exp Metastasis. 2015 Jun 27;32(6):605–22. doi: 10.1007/s10585-015-9731-4 (PMC4503866; doi:10.1007/s10585-015-9731-4)
Supplement: Supplementary file 2 — Supplementary material 2 (DOCX 74 kb) [file 10585_2015_9731_MOESM2_ESM.docx]

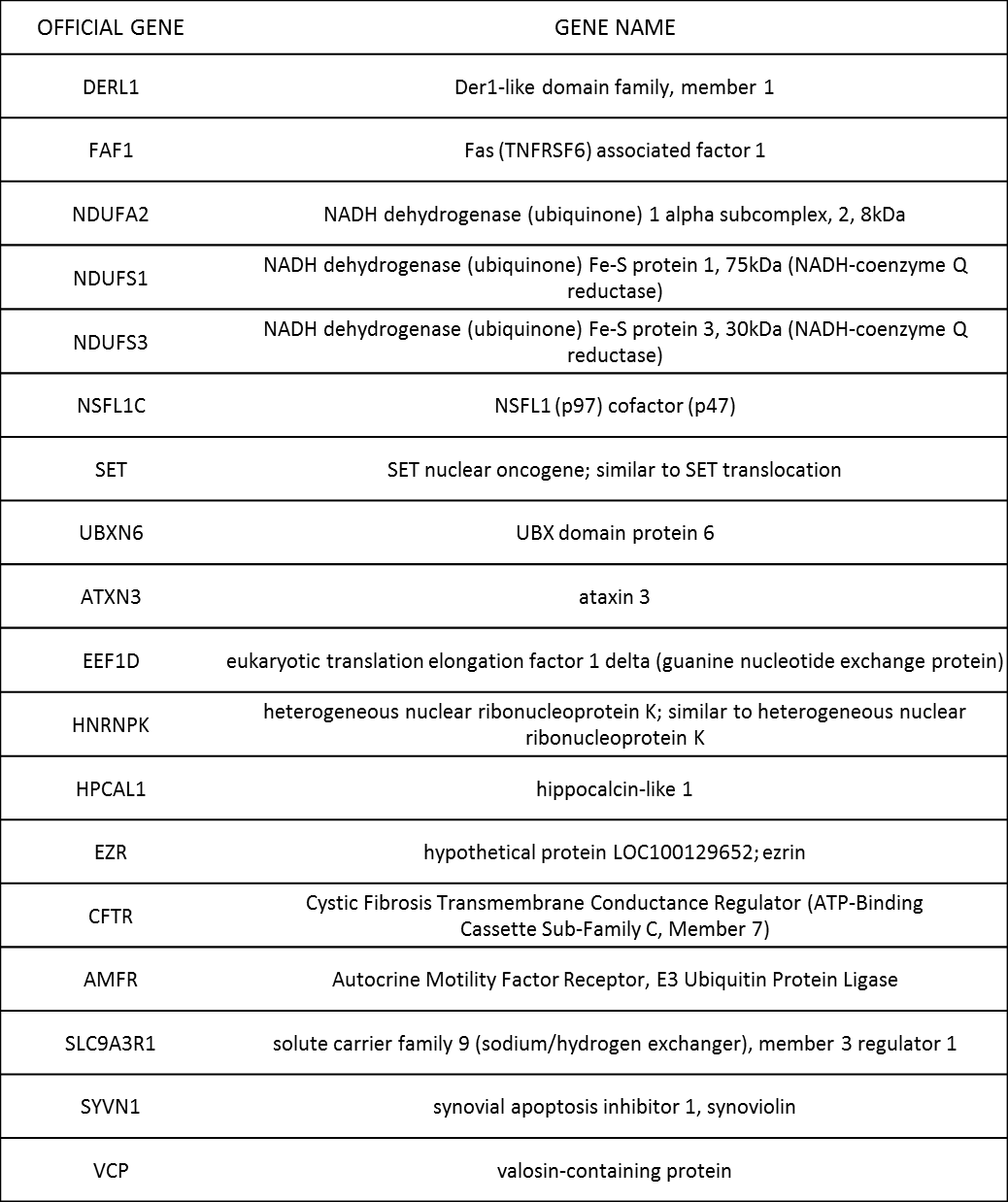


**Supplementary 3 (Table1).** According to protein-protein interaction in STRING, the names of 8+10 genes corresponding to all proteins were list.
