# Supplementary material for: Eight proteins play critical roles in RCC with bone metastasis via mitochondrial dysfunction
Source: Clin Exp Metastasis. 2015 Jun 27;32(6):605–22. doi: 10.1007/s10585-015-9731-4 (PMC4503866; doi:10.1007/s10585-015-9731-4)
Supplement: Supplementary file 3 — Supplementary material 3 (DOCX 66 kb) [file 10585_2015_9731_MOESM3_ESM.docx]

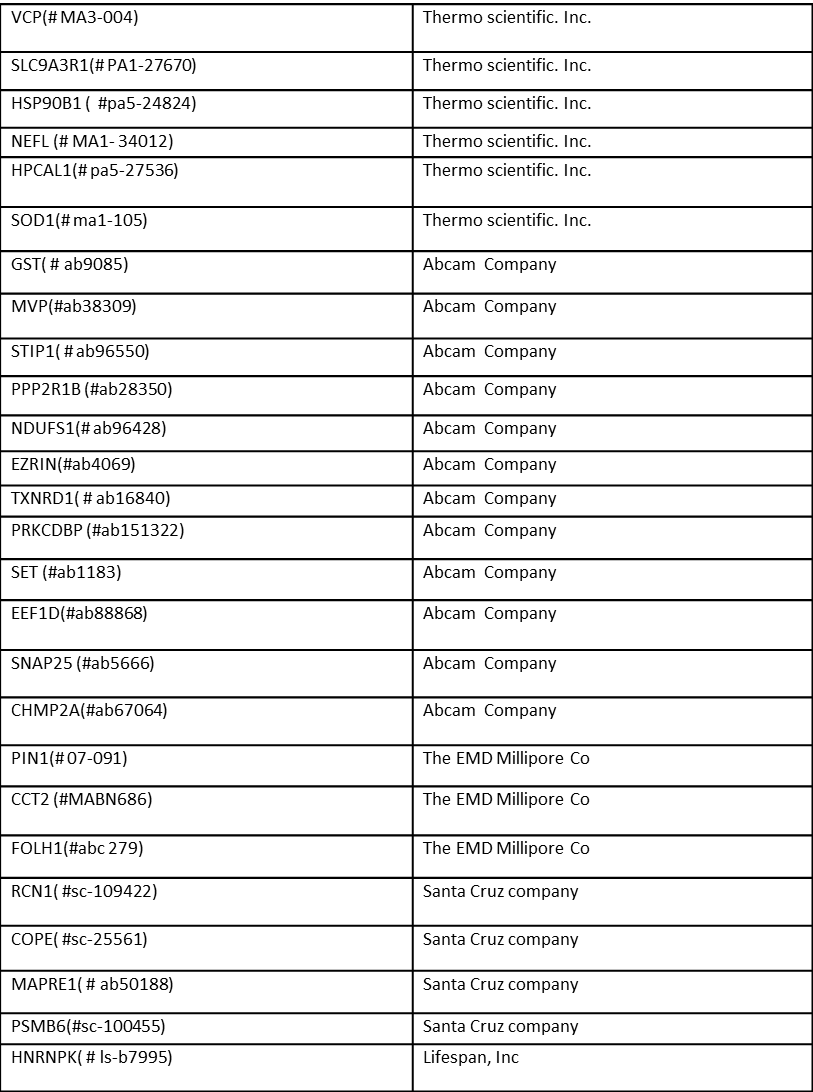


**Supplementary 4 (Table2).** The primary antibodies resources were listed, which were used in western blotting and immunohistochemistry assay.
