# Supplementary material for: Eight proteins play critical roles in RCC with bone metastasis via mitochondrial dysfunction
Source: Clin Exp Metastasis. 2015 Jun 27;32(6):605–22. doi: 10.1007/s10585-015-9731-4 (PMC4503866; doi:10.1007/s10585-015-9731-4)
Supplement: Supplementary file 4 — Supplementary material 4 (DOCX 107 kb) [file 10585_2015_9731_MOESM4_ESM.docx]

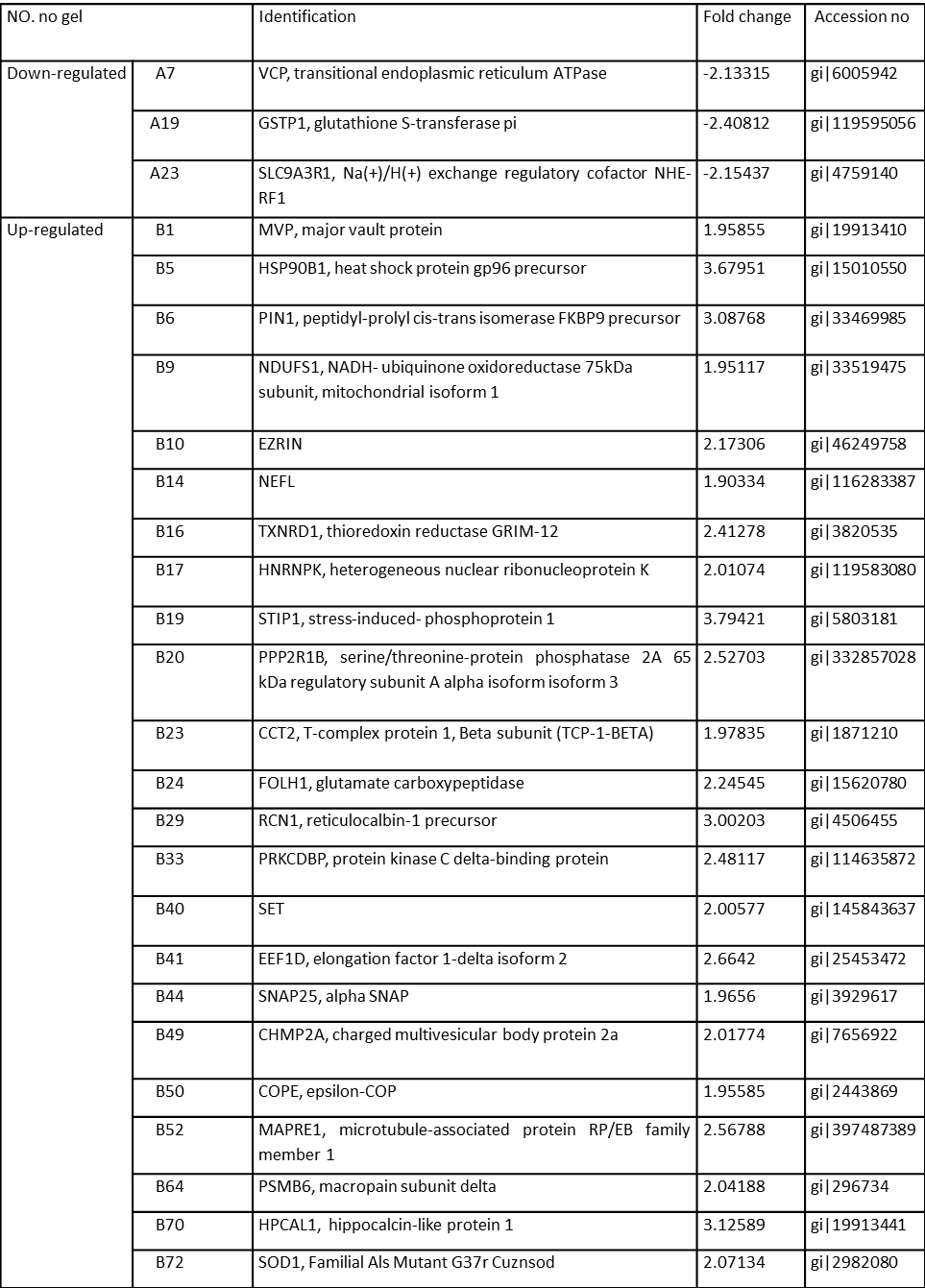


**Supplementary 5 (Table 3).** The final identified 26 proteins from 2-DE include 3 down-regulated and 23 up-regulated proteins.
